# Skin cancers arising within tattoos: A systematic review

**DOI:** 10.1016/j.jdin.2024.03.015

**Published:** 2024-04-20

**Authors:** Jamie Lebhar, Jennifer Jacobs, Chandler Rundle, Samantha J. Kaplan, Paul J. Mosca

**Affiliations:** a Duke University School of Medicine, Durham, North Carolina; b Department of Dermatology, Duke University Health System, Durham, North Carolina; c Duke University Medical Center Library & Archives, Duke University School of Medicine, Durham, North Carolina; d Division of Surgical Oncology, Duke University Health System, Durham, North Carolina

**Keywords:** basal cell carcinoma, keratoacanthoma, melanoma, nonmelanoma skin cancers, skin cancer, squamous cell carcinoma, tattoos

## Abstract

**Background:**

Tattooing is a widespread practice and has increased in popularity over time. Many lesions have been described in relation to tattoos, including malignant tumors.

**Objectives:**

The primary goal of this review is to determine whether the frequency of published cases of skin cancers within tattoos has been increasing over time.

**Methods:**

Our review is in adherence to the Preferred Reporting Items for Systematic Reviews and Meta-Analyses guidelines and reporting criteria. The databases MEDLINE via PubMed, Embase via Elsevier, and Scopus via Elsevier were searched from inception to February 23, 2023. No data or publication date limits were imposed.

**Results:**

Our review identified 160 cases of cutaneous tumors arising within tattoos. An increase in published cases over time was observed. Most reported tumors developed within red tattoo pigment (36.9%), with the largest contribution by squamous cell carcinoma and keratoacanthoma lesions.

**Limitations:**

There was a lack of consistency of information in published case reports which limited the scope of our analysis. Small sample size was also a limitation of this review.

**Conclusions:**

With the increased popularity of tattoos, it is helpful to continue reporting cases of cutaneous malignancies within tattoos. Awareness of the frequency and severity of tumors within tattoos may be communicated to the public.

**Capsule Summary**

• There are published reviews of skin cancers arising in tattoos; however, there is not a recently published rigorous systematic review.
• Our review shows an increase in published cases of cutaneous malignancies in tattoos over time. Further research is necessary to evaluate a potential association between tattoos and cutaneous malignancies.

## Introduction

Tattooing is body modification through introduction of exogenous pigments and dyes into the dermis of skin.^1^ The tattooing industry has been growing since the 1970s with a particularly rapid increase since the early 2000s.^1^, ^2^, ^3^ The prevalence of tattoos in the United States is approximately 46% of the general population, with a large contribution from individuals under the age of 45.^2^^,^^4^ A recent poll showed that among generations in America, millennials, those born between 1981 and 1996, had the greatest percentage of individuals with 1 or more tattoos as of 2021, followed by Gen X and Gen Z.^5^ Also, 35% to 48% of survey participants in European countries including the United Kingdom, Germany, France, Spain, and Denmark report having at least 1 tattoo.^4^

With the increased popularity of tattoos, there have also been several reports of adverse events after tattooing. Reported complications include infections, allergic reactions, and benign and malignant tumors. Although rare cutaneous malignancies of tattooed skin have included malignant melanoma, squamous cell carcinoma, basal cell carcinoma, keratoacanthoma, lymphoma, and other tumors.^6^ Although there are numerous reports of skin cancer arising in tattoos, it is unknown if there has been an increase in the number of cases published over time. It is also unclear if there is a causal relationship between tattoos and the development of cutaneous malignancies.

## Methods

### Data sources

**Fig 1:**
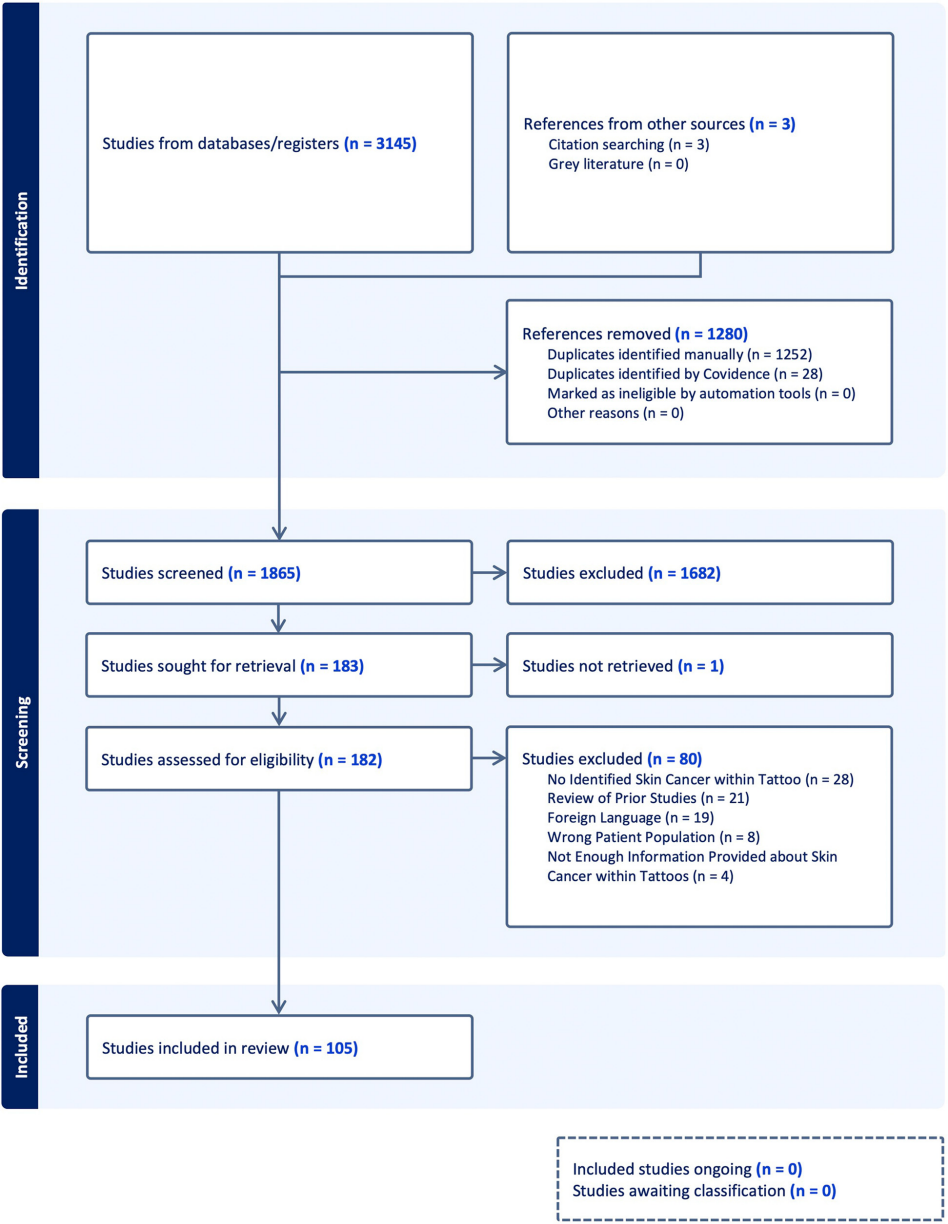
Preferred reporting items for systematic reviews and meta-analyses diagram.

We conducted our systematic review following the Preferred Reporting Items for Systematic Reviews and Meta-Analyses guidelines and reporting criteria. Our search strategies were developed with the help of a medical librarian (S.K.). We searched for original primary articles on patients with cutaneous malignancies arising within a tattoo. We performed a systematic search of all published primary articles within the following databases: PubMed/Medline, EMBASE, and Web of Science. Reference lists of eligible studies were also reviewed to identify additional publications. If an article was added outside of the 3 original databases, we included a box in the Preferred Reporting Items for Systematic Reviews and Meta-Analyses diagram for documentation (Fig 1). We utilized Covidence software for screening studies in our systematic review and there were no data limits on the search.

### Study selection

Citations from all search results were downloaded and merged using a reference management software package (EndNote). Our authors (J.L., J.J.) screened study titles and abstracts for potential inclusion and reviewed full-text articles, including reference lists, to determine their eligibility. Study types that were eligible included case reports, case series, letters to the editor, retrospective observational studies, photo challenges, and abstracts. Studies were eligible for inclusion if they demonstrated a case in which skin cancer arose within tattooed skin. There were no publication date limits on eligibility. Exclusion criteria included lesions arising on tattooed mucosa. Also, articles that were not available in English and articles that did not describe skin cancer developing within tattooed skin were excluded. Author J.L. collected the data from each eligible report and performed data analysis and created figures on excel.

## Results

### Study characteristics

**Fig 2:**
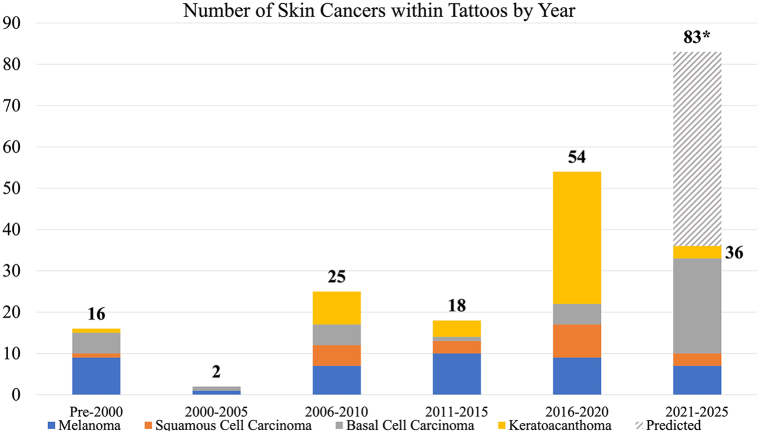
Timeline of skin cancer cases arising within tattooed skin grouped by tumor type. ∗ Includes predicted data for the years 2023 to 2025 based on trending number of cases from 2021 to February 2023.

**Fig 3:**
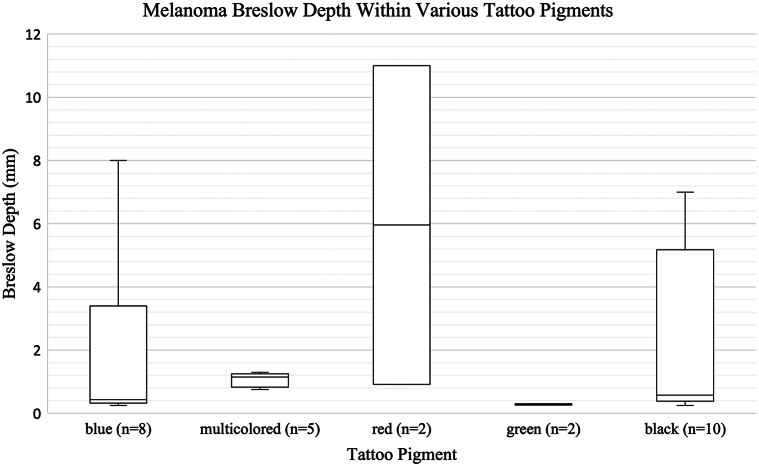
Reported melanoma Breslow depth within various tattoo pigments. The number value shown is the median Breslow depth of the melanoma lesion within the respectively indicated tattoo pigment. The *top* and *bottom* of the box represent the upper quartile (the median of the *upper* values) and lower quartile (the median of the *lower* values). The *lines* outside the box extend to the maximum and minimum Breslow depth values.

**Fig 4:**
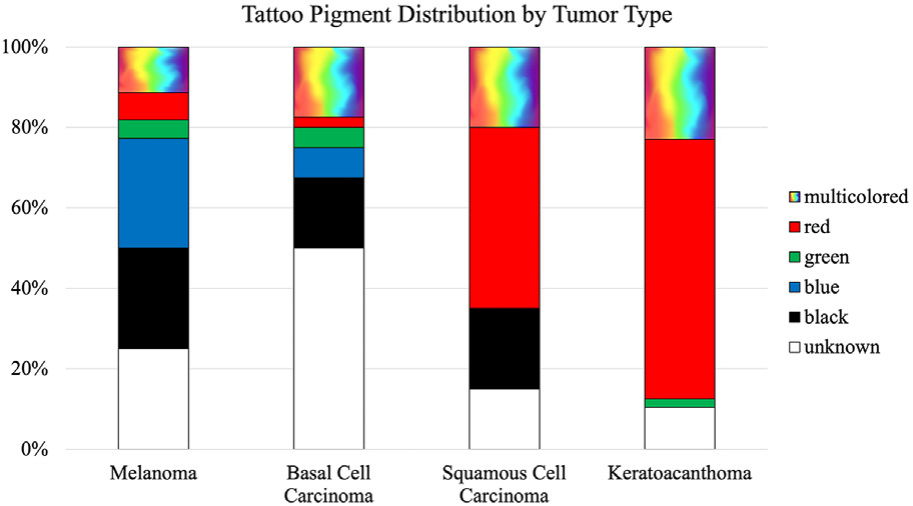
Tattoo pigment in which the specified tumor type developed in. Melanoma lesions predominated in *blue* and *black* tattoo pigments. Basal cell carcinoma developed most frequently in *black* and multicolor tattoo pigments. The majority of squamous cell carcinoma and keratoacanthoma lesions developed within *red* tattoo pigment.

Of the 1868 unique citations identified by our search, 105 studies with 160 patients met the eligibility criteria (Fig 1). Of the 105 studies, there were 70 case reports, 2 case series, 18 letters to the editor, 2 photo challenges, 4 retrospective observational studies, and 9 abstracts. The reported cases of skin cancer within tattoos were organized by publication date in Fig 2. Among the published cases of cutaneous malignancies developing within tattoos, information was not described for 29 patients regarding gender, 31 patients regarding location of lesion, 70 patients regarding treatment, 8 patients regarding melanoma Breslow depth, and 41 patients regarding tattoo pigment color (Table I, Figs 3 and 4).

**Table I:** Reported cases of melanoma, basal cell carcinoma, squamous cell carcinoma, keratoacanthoma, and other tumors arising within tattooed skin

Tumor type/cases	Gender	Average age/range	Location		Treatment		Dates published
Melanoma 43 cases	Male- 81.4% (35)	46 (9-82)	Face	2.3% (1)	Excision	32.6% (14)	1938-2022
			Chest	9.3% (4)	Excision and LND	11.6% (5)	
	Female- 7% (3)	41 (34-54)	Abdomen	4.7% (2)	Excision and SLNBx	6.9% (3)	
			Back	16.3% (7)	Multiple excisions	6.9% (3)	
			Arm	51.2% (22)	Excision and other therapies	4.7% (2)	
	Unknown- 11.6% (5)	-	Leg	6.9% (3)	Unknown	37.2% (16)	
			Unknown	9.3% (4)			
Basal cell carcinoma 40 cases	Male- 35% (14)	54 (40-66)	Face	15% (6)			1976-2023
			Chest	-	5-FU ointment	2.5% (1)	
	Female- 25% (10)	49 (28-72)	Abdomen	-	Excision	22.5% (9)	
			Back	25% (10)	MMS	12.5% (5)	
			Arm	22.5% (9)	Unknown	62.5% (25)	
	Unknown- 40% (16)	35	Leg	-			
			Unknown	37.5% (15)			
Squamous cell carcinoma 20 cases	Male- 50% (10)	50 (35-79)	Face	9.5% (2)∗			1966-2022
			Chest	-	Excision	45% (9)	
	Female- 40% (8)	50 (24-74)	Abdomen	-	MMS	20% (4)	
			Back	-	Multiple excisions	5% (1)	
			Arm	38.1% (8)∗	Excision and other therapies	5% (1)	
	Unknown- 10% (2)	-	Leg	33.3% (7)	Unknown	25% (5)	
			Unknown	19% (4)			
Keratoacanthoma 48 cases	Male- 44.8% (26)	49 (24-70)			Excision(s)	22.6% (12)	1973-2021
			Face	2.1% (1)	MMS	3.8% (2)	
			Chest	-	Diflucortolone valerate	3.8% (2)	
	Female- 33.3% (16)	46 (24-65)	Abdomen	-	5-FU ointment	1.9% (1)	
			Back	2.1% (1)	Acitretin	11.3% (6)	
			Arm	29.2% (14)	Intralesional MTX or Kenalog	7.5% (4)	
	Unknown- 10.3% (6)	-	Leg	52.1% (25)	Biopsy ± electrodessication	3.8% (2)	
			Unknown	14.6% (7)	Laser or photo therapy	3.8% (2)	
					Unknown or no treatment	41.5% (22)	
Dermatofibrosarcoma protuberans 4 cases	Male- 50% (2)	44 (35-52)	Back	50% (2)	Multiple excisions	50% (2)	2005-2020
	Female- 50% (2)	33 (29-37)	Arm	25% (1)	MMS	50% (2)	
	Unknown- 0	-	Leg	25% (1)			
Dermatomyofibroma 1 case	Male	26	Chest		Unknown		2013
Leiomyosarcoma 1 case	Male	41	Arm		Excision		2008
Desmoplastic iSpitz nevus 1 case	Female	28	Arm		Excision		2018
Non-Hodgkins lymphoma 1 case	Male	32	Arm		Excision		1978
B-cell lymphoma 1 case	Male	54	Arm		Unknown		1992

*5-FU*, 5 Fluorouracil; *LND*, lymph node dissection; *MMS*, Mohs micrographic surgery; *MTX*, methotrexate; *SLNBx*, sentinel lymph node biopsy.

∗ One case reported SCC lesion occurred on face and arm.^7^

### Study appraisals

Evaluating the articles by Joanna Briggs Institute critical appraisal checklists for case reports (J.L., J.J.) showed that 91.2% published studies met the conditions by a proportion of at least 60% compatibility (Supplementary Table I, available via Mendeley at https://data.mendeley.com/datasets/j8mz43t6rg/1). This proportion was not met for 2 case reports, 1 letter to the editor, 5 retrospective observational studies, and 2 abstracts; however, they were not excluded due to meeting the necessary inclusion criteria required for our study. Limited numbers of published cases of skin cancers occurring within tattoos constrained us to include any study type that described this event.

### Reported skin cancers over time

A total of 160 cases of skin cancer arising within tattoos have been published in the literature since 1938 (Fig 2). From 1938 through 2006, there have been occasional published cases of cutaneous malignancies in tattoos, ranging from zero to 3 cases per year. There were 7 reported cases in 2007 which marked the beginning of an increasing trend, albeit with significant fluctuations. The highest number of cases occurred in 2020 and 2021 with 29 and 31 published cases respectively. The 5-year total for the period 2016 to 2020 was 54, and the annualized projection for 2021 to 2025 based on the previously published reports is 83 cases (Fig 2).

### Melanoma

A total of 43 cases of melanoma arising within a tattoo were identified.^6^, ^8^, ^9^, ^10^, ^11^, ^12^, ^13^, ^14^, ^15^, ^16^, ^17^, ^18^, ^19^, ^20^, ^21^, ^22^, ^23^, ^24^, ^25^, ^26^, ^27^, ^28^, ^29^, ^30^, ^31^, ^32^, ^33^, ^34^, ^35^, ^36^, ^37^, ^38^, ^39^, ^40^, ^41^ The majority of cases occurred in males (81.4%) (Table I). Most of the malignances were located on the arm (51.2%), followed by the back (16.3%) (Table I). Although most of the lesion’s subtypes were unclassified or unknown (53.5%), the most common type specified was superficial spreading melanoma (37.2%), followed by nodular melanoma (9.3%). The median Breslow depth was 0.91 mm and the average Breslow depth was 2.86 mm. The median melanoma Breslow depth was highest (5.96 mm) in lesions arising within red tattoo pigment (Fig 3). Melanoma predominantly developed in blue (27.9%) and black tattoo pigment (25.6%) (Fig 4). The lesions were treated with excision, and additional therapy (lymph node dissection, sentinel lymph node biopsy, multiple excisions, and adjuvant therapy) was required in select cases (Table I).

### Basal cell carcinoma

A total of 40 cases of basal cell carcinoma arising in a tattoo were identified.^6^, ^39^, ^42^, ^43^, ^44^, ^45^, ^46^, ^47^, ^48^, ^49^, ^50^, ^51^, ^52^, ^53^, ^54^, ^55^, ^56^, ^57^, ^58^ The cases were reported most frequently in males (35%) followed by females (25%) (Table I). The majority of the lesion’s body location was unknown (37.5%) followed by back (25%) (Table I). The most common subtypes diagnosed were nodular (17.5%) or superficial (17.5%) followed by mixed superficial and nodular (5%) (Table I). Basal cell carcinoma predominantly developed in blue (35%) and multicolored tattoo pigment (35%) (Fig 4). In most cases, the treatment modality was not reported (62.5%); however, among reported treatments were excision, Mohs micrographic surgery, and 5-fluorouracil (Table I).

### Squamous cell carcinoma

A total of 20 cases of squamous cell carcinoma arising in a tattoo are described in the literature.^7^, ^39^, ^59^, ^60^, ^61^, ^62^, ^63^, ^64^, ^65^, ^66^, ^67^, ^68^, ^69^, ^70^, ^71^, ^72^, ^73^, ^74^ The cases were reported most frequently in males (50%) followed by females (40%) (Table I). Most of the lesions were located on the extremities (71.4%) (Table I). In particular, in 1 case a patient noticed the lesion of concern after undergoing multiple laser tattoo removal treatments.^59^ Notably, the majority of lesions occurred within red tattoo ink (55% in red and multicolor including red) (Fig 4). The lesions were treated with excision or Mohs micrographic surgery, and additional therapy (multiple excisions, imiquimod, and 5-fluorouracil cream) was required in select cases (Table I).

### Keratoacanthoma

A total of 48 cases of keratoacanthoma arising in a tattoo are noted in the literature.^7^, ^62^, ^65^, ^71^, ^75^, ^76^, ^77^, ^78^, ^79^, ^80^, ^81^, ^82^, ^83^, ^84^, ^85^, ^86^, ^87^, ^88^, ^89^, ^90^, ^91^, ^92^, ^93^, ^94^, ^95^, ^96^, ^97^, ^98^, ^99^, ^100^, ^101^, ^102^, ^103^, ^104^, ^105^, ^106^, ^107^, ^108^, ^109^, ^110^ The cases were reported most frequently in males (44.8%) followed by females (33.3%) (Table I). Most of the lesions were located on the extremities (81.3%) (Table I). In particular, 1 case noticed lesions develop after undergoing a second treatment of laser tattoo removal.^75^ As in the case of squamous cell carcinoma, the majority of keratoacanthoma lesions occurred within red tattoo ink (79.2%, red and multicolor including red) (Fig 4). The lesions were treated with a wide variety of modalities, including excision(s), Mohs micrographic surgery, diflucortolone valerate, 5-fluorouracil, acitretin, intralesional methotrexate or triamcinolone, biopsy and/or electrodessication, laser therapy, and photodynamic therapy (Table I).

### Other tumors

A total of 9 miscellaneous tumors arising in a tattoo are described in the literature.^77^, ^78^, ^79^, ^84^, ^88^, ^89^, ^90^, ^91^, ^93^, ^97^ There were 4 cases of dermatofibrosarcoma protuberans, 1 dermatomyofibroma, 1 leiomyosarcoma, 1 desmoplastic intradermal Spitz nevus, 1 non-Hodgkin lymphoma, and 1 B-cell lymphoma arising within tattoos. Most of the cases occurred in males (66.7%, 6) (Table I). Most of the lesions were located on the arm (55.5%) followed by the back (22.2%) (Table I). For cases in which treatment modality was reported, treatments included surgical excision or Mohs micrographic surgery (Table I).

## Discussion

This systematic review of cutaneous malignancies arising within tattoos is the largest compilation of cases to date.^6^ This review shows an increase in published cases of cutaneous malignancies within tattoos over time. From 2016 to 2023, there have been nearly twice the number of cases published since the reporting period of 1938 to 2015. The annualized projection for 2021 to 2025 is 86 cases, which would be the most cases reported in any 5-year period. Further investigation is warranted to establish an accurate estimate of the actual incidence of malignancies arising within tattooed skin.

There are noteworthy differences of patient and tumor characteristics for melanoma within tattoos versus nontattooed skin. Melanoma within tattoos was reported more frequently in males (81.8%) as compared to cutaneous melanomas (56.9%).^111^ Further, individuals with melanoma in tattoos were diagnosed younger (45.2 years) than melanoma in nontattooed skin (60.6 years).^111^ The median and average Breslow depth (0.91 mm, 2.86 mm) for melanomas within tattoos were reported to be higher than the melanoma thickness trends in the general population (0.58-0.73 mm, 0.64-0.77 mm).^112^ Tattooed individuals might pay closer attention to their tattoo’s appearance, thus identifying concerning lesions earlier. Alternatively, tattoos may predispose individuals to the development of cutaneous malignancies, resulting in early skin cancers. Particularly, the higher Breslow depths seen in melanomas in tattoos do not support the idea that tattoos result in earlier detection of melanoma. Notably, the distributions of characteristics reported in this study do not represent a surrogate for a population-based study.

Although black pigment is considerably the most utilized tattoo color, our review illustrates that skin cancers developed predominantly within other tattoo colors.^113^ The greatest melanoma Breslow depth was reported in red pigment followed by multicolored tattoos. Notably, many melanoma lesions developed dark tattoo pigments (blue and black); these darker pigments may mask the concerning lesions and potentially lead to delays in skin cancer diagnosis for tattooed patients.

There were also notable findings for nonmelanoma skin cancers arising within tattoos. Nonmelanoma skin cancers most frequently occur in sun-exposed sites; however, basal cell carcinoma in tattooed skin interestingly was highly reported on the back. It could be speculated that tattoos provoke or predispose individuals to developing nonmelanoma skin cancer, potentially in uncommon locations. Further, most squamous cell carincoma and keratoacanthoma lesions predominated within red ink. It has been reported that red 22 tattoo ink is cleaved when exposed to ultraviolet (UV)-B radiation or natural sunlight, and the cleavage products are hazardous with risk of toxicity or carcinogenicity.^114^ Further, a study investigating red tattoos on mice exposed to UV radiation showed faster squamous cell carincoma tumor onset when compared to control mice without tattoos.^115^ In the United States, the production of tattoo ink is unregulated and there are no standard guidelines issued by national agencies.^116^ Further research is warranted to screen for the presence of toxic chemicals in tattoo ink.^117^

There is speculation regarding the pathophysiology of cancer arising within tattooed skin. Some postulate that trauma resulting from ink injection may induce inflammation and damage skin cells resulting in melanocyte mutations.^53^ Also, tattoo pigment may alter the absorption of UV radiation resulting in increased susceptibility for tumor development.^53^ Malignancy may occur from increased UV radiation and DNA damage or hazardous cleavage byproducts of tattoo pigments.^114^^,^^118^ Further, some components within tattoo pigment have been revealed as carcinogenic. Additives in tattoo ink have changed over time and examples of components that are carcinogenic or genotoxic include titanium dioxide nanoparticles, benzo(a)pyrene, cadmium compounds, benzo(a)antrahcene, benzo(k)fluoranthene, benzo(b)fluoranthene, chrysene, naphthalene, mercury, and soluble cobalt salt.^93^

Lesions presenting within a tattoo may present a diagnostic challenge. Tattoos can camouflage the appearance of skin cancer, leading to delayed detection. This masking of suspicious lesions creates difficulties in assessment macroscopically and microscopically. Dermatoscopic assessment may be impeded by ink altering the appearance of clinical signs such as distribution of pigment, vascular morphology, and borders of the lesion. For pathologists, the deposition of tattoo pigment within tissue specimens may hamper the diagnostic process for either other dermatoses or a lymph node metastasis. Health care practitioners must be aware of tattoo-associated complications and must maintain a high level of vigilance in screening and monitoring of lesions and interpreting biopsy specimens.

Physicians should maintain high clinical suspicion for new and growing lesions arising within tattooed skin. Tattoos should be screened regularly during skin exams and throughout laser tattoo removal. It has been shown that laser therapy on malignant cells in vitro has increased proliferation of cancer cells.^119^ Thus, if a concerning lesion is identified within the laser site, biopsy should be advised and laser tattoo removal should be withheld until appropriate treatment is completed. It would be beneficial for dermatologist to perform laser tattoo removal to provide screening skin exams throughout laser sessions.

Patients at increased risk of developing skin cancer (eg, those with multiple atypical nevi, xeroderma pigmentosum, family history of skin cancer, or immunosuppression) should be advised by the tattoo community and health professionals to first undergo dermatologic assessment before acquiring a tattoo. Additionally, tattoos should never be placed over preexisting melanocytic nevi or premalignant lesions. Nevi that are covered by tattoos may change in appearance and go unnoticed, resulting in delayed diagnosis of malignancies.

Several limitations of our systematic review must be acknowledged. Although this review illustrated an increase in published cases of skin cancers within tattoos over time, this increase is not a surrogate for incidence over time. Further, the case reports lacked consistency in reporting background information on patient history of sun exposure and skin cancers. Information about stage and other tumor characteristics was frequently not available, consequently limiting the scope of our analysis. Also, several reports did not provide information of tattoo pigment color in which the skin cancer developed and/or images of the tattoo. Sample size was also a limiting factor in our review. Of the studies that met the eligibility requirements, there were numerous reports that were excluded due to only being available in a foreign language. Due to small sample size and inconsistent reporting information by study, statistical analysis was limited to descriptive statistics.

This review is the largest compilation of studies of cutaneous malignancies arising within tattoos and shows an increasing number of published cases of skin cancers within tattoos. Tattooists and dermatologists should be mindful of safe tattoo practices, educate individuals on adverse reactions in tattoos, ensure close monitoring of tattooed skin, and refer/provide appropriate treatment when necessary.

## Conclusions

This systematic review determined that the frequency of published cases of skin cancers arising within tattoos have been increasing over time. Additionally, various patient and tumor characteristics in these cases were different when compared to the general population. These findings raise the question of whether tattoos may influence the development, detection, and biology of cutaneous malignancies in tattooed skin. Further investigation is warranted to explore these potential relationships.

## Conflicts of interest

None disclosed.
